# Histological Criteria for Encapsulating Peritoneal Sclerosis – A Standardized Approach

**DOI:** 10.1371/journal.pone.0048647

**Published:** 2012-11-07

**Authors:** Niko Braun, Peter Fritz, Christoph Ulmer, Joerg Latus, Martin Kimmel, Dagmar Biegger, German Ott, Fabian Reimold, Klaus-Peter Thon, Juergen Dippon, Stephan Segerer, M. Dominik Alscher

**Affiliations:** 1 Department of Internal Medicine, Division of Nephrology, Robert Bosch Hospital, Stuttgart, Germany; 2 Margarete Fischer–Bosch Institute of Clinical Pharmacology, University of Tuebingen, Tuebingen, Germany; 3 Department of Surgery, Robert Bosch Hospital, Stuttgart, Germany; 4 Department of Diagnostic Medicine, Division of Pathology, Robert Bosch Hospital, Stuttgart, Germany; 5 Department of Internal Medicine, Beth Israel Deaconess Medical Center, Boston, Massachusetts, United States of America; 6 Department of Mathematics, University of Stuttgart, Stuttgart, Germany; 7 Division of Nephrology, University Hospital, Zurich, Switzerland; Queensland Institute of Medical Research, Australia

## Abstract

**Background:**

The two most relevant pathologies of long-term peritoneal dialysis (PD) are simple sclerosis and encapsulating peritoneal sclerosis (EPS). The histological differentiation of those two entities is difficult. The Aim of the study was to establish a method to standardize and facilitate the differentiation between simple sclerosis and EPS

**Methods:**

We investigated 58 peritoneal biopsies - 31 EPS patients and 27 PD patients. Two blinded investigators analyzed 20 histological characteristics in EPS and PD patients.

**Results:**

The following findings were significantly more common in EPS than in patients on PD without EPS: fibroblast like cells (FLC) (p<0.0001), mesothelial denudation (p<0.0001), decreased cellularity (p = 0.008), fibrin deposits (p<0.03), Fe deposits (p = 0.05), podoplanin vascular (p<0.0001), podoplanin avascular (p<0.0001). Using all predictor variables we trained the classification method Random Forest to categorize future cases. Podoplanin vascular and avascular were taken together (p<0.0001), FLC (p<0.0001), mesothelial denudation (p = 0.0005), calcification (p = 0.0026), acellular areas (p = 0.0094), and fibrin deposits (p = 0.0336) showed up as significantly important predictor variables. Estimated misclassification error rate when classifying new cases turned out to be 14%.

**Conclusion:**

The introduced statistical method allows discriminating between simple sclerosis and EPS. The misclassification error will likely improve with every new case added to the database.

## Introduction

Encapsulating peritoneal sclerosis (EPS) is a rare but devastating complication of peritoneal dialysis (PD). Although several medical and surgical treatment approaches exist, morbidity and mortality are still high [1]–[3]. Clinical symptoms, radiologic findings, and histological criteria are the three diagnostic pillars of EPS [4].

If clinical signs of bowel obstruction, abdominal pain or weight loss occur, the disease is probably in an advanced stage. Earlier signs, including changes in transporter status or ultrafiltration failure, are common but not specific for the disease [5]–[9].

Even though abdominal computed tomography (CT) scanning is an established diagnostic tool in EPS and the existing imaging criteria are well defined, CT scanning alone does not allow one to make the diagnosis of EPS. All the described imaging features were also found in patients on PD without clinical signs of EPS [10]–[13].

The two most relevant pathologies of long-term PD are simple sclerosis and EPS. PD-induced simple fibrosis of the peritoneal membrane is a very common finding in PD-patients as previously described in the literature [14], [15]. Compared to PD patients, histological findings in EPS are described but not specific for the disease [16]. Some authors state, that EPS is always a result of long term PD, dependent on the duration of PD treatment. Others state, that for the development of EPS a so called second hit is mandatory and that PD duration itself does not cause EPS. Discussed second hits are a severe peritonitis, an increased peritonitis rate, several PD fluids or cessation of PD treatment [17], [18]. For a histological diagnosis of EPS, it is mandatory to define reproducible histological criteria that can be used to differentiate the two entities [6].

There are several publications about biopsy techniques, tissue preparation and histological findings of the peritoneum [14], [18]–[21]. For immunohistochemistry, the method of cutting the paraffin embedded tissue is not of great importance. But for morphological assessment, including the thickness of the submesothelial cell layer or the extent of fibrosis, it is essential that histology techniques are standardized. On the other hand, it is important to keep techniques simple. Therefore, cutting the tissue perpendicular to the surface could be feasible. Due to technical reasons (small operational access) a trauma-free removal of peritoneum is often impossible and obtained tissue samples are sometimes to small for pinning onto cork boards.

In 2003 and 2005 Honda and colleagues published a paper with 12 EPS patients, which showed that fibrin deposition, fibroblast swelling, capillary angiogenesis and mononuclear cell infiltration were significantly more common in EPS than in peritonitis, ultrafiltration failure, uremia and so called “pre-EPS”. Regarding the degree of these parameters, only fibroblast swelling and fibrin deposition exhibited statistically significant differences [20]. Several markers for fibroblast proliferation were also investigated.

Garosi and colleagues investigated 224 peritoneal biopsies of non-EPS patients and compared the morphological findings with the biopsies of 39 patients with EPS. Significant findings in patients with EPS were thickening of the submesothelial cell layer, vasculopathy, inflammation, arterial occlusion, tissue calcification and ossification and arterial calcification and ossification [22].

In 2008 Sherif and colleagues compared 12 EPS patients with 23 non-EPS patients. Only fibrin deposition and the thickness of the compacta were significantly different [21].

The problem associated with most of the published data is that data acquisition was not standardized, observers were not blinded to the diagnosis, intra- and inter-observer variability was not given. Up to now there is no established method to differentiate between EPS and simple sclerosis.

The first aim of our study was to define relevant and reproducible histological parameters in combination with an immunohistochemical parameter in patients with EPS compared to patients on PD without EPS [16]. Secondly, we tried to establish a statistical method to standardize and facilitate the differentiation of simple sclerosis and EPS for new cases.

## Materials and Methods

Biopsies from the peritoneum were formalin-fixed in 4% buffered formalin and paraffin-embedded following routine protocols [23]. Included were biopsies from 27 patients on PD without signs of EPS and 31 patients with clinical and radiological diagnostic criteria for EPS [5], [12]. Biopsies were taken from patients on PD at the time of catheter removal, correction of a catheter malposition or during abdominal surgery (e.g. hernia repair, cholecystectomy). Patients with an episode of peritonitis within the last 6 months were excluded. The biopsies from patients with EPS were taken at the time of peritonectomy. Clinically, all patients were in a late stage of the disease with recurrent abdominal pain caused by chronic bowel obstruction.

All patients had given their informed consent regarding a scientific work-up of tissues taken during routine procedures.

Immunohistochemistry was performed as previously described [24], [25]. Dewaxed and rehydrated tissue sections were incubated in Peroxidase Blocking Solution (S 2023, DAKO, Hamburg, Germany) (to block endogenous peroxidases). Pretreatment was performed in a steamer, using an antigen retrieval solution (pH 9, S 2367, DAKO). For immunostaining we used a Techmate system (TechMate 500 Plus, DAKO). The staining method used a dextran-coated peroxidase coupled polymer system (Dako REAL™ EnVision™ Detektion Kit, Peroxidase/DAB+, Rabbit/Mouse, K 5007, DAKO).

A monoclonal mouse antihuman podoplanin antibody (D2–40, DAKO, M 3619) was used on all biopsies, diluted 1∶100 in a commercial buffer system (antibody diluent, DAKO, S 2022) [16]. As positive control, we used tissue with lymphatic vessels. A negative control specimen was created by omitting the primary antibody. Podoplanin was evaluated as either vascular or podoplanin avascular (0,1,2,3).

From each slide hematoxylin and eosin staining was done for morphological analysis: fibrosis: absent, 1–10%/low-power field (LPF), 11–50%/LPF, >51%/LPF (0,1,2,3); a additional quantitative consensus evaluation of the degree of fibrosis was done. Tissue sections were scanned, visualized and the thickness of the fibrosis zone was measured using the software program Image Manager, version 4.0, Leica, Germany. FLC: absent, 1/5 high-power fields (HPFs), 2–4/5 HPFs, >5/5 HPFs (0,1,2,3); exudation: absent, 1 small area in 1 MPF, 1 area <50%/MPF, 1 area >50%/medium-power field (MPF) (0,1,2,3); cellularity was evaluated as 0 (1–2 nuclei/HPF), 1 (3–5 nuclei/HPF) 2 (6–20 nuclei/HPF) and 3 (>20 nuclei/HPF); variability of cellularity: absent or present (0,1); vessel density: absent, 1–5/HPF, 6–10/HPF, >10/HPF in the submesothelial cell layer and variability of vessel density as absent or present (0,1); acute inflammation (neutrophiles): absent, 1/HPF, 2–5/HPF, >5/HPF (0,1,2,3); chronic inflammation (round cells): absent, 1–5/HPF, 6–20/HPF, >20/HPF (0,1,2,3); hemorrhage: absent extravasal erythrocytes, 1 area <10%/5 LPF, 2+3 area/5 LPF or 1 area 11–30%/LPF, 4+5 area/5 LPF or 1 area >30%/LPF (0,1,2,3); mesothelial hyperplasia: more than 2 layers of mesothelial cells (0,1); fibrin deposits: absent eosinophilic area, 1 area <5%/5 MPF, 1 area 6–20%/5 MPF, 1 area >20%/5 MPF (0,1,2,3); presence of vasculopathy: thickening of vessel walls and/or inflammation of the vessel wall (0,1); mesothelial denudation: no visible mesothelium (0,1); presence of acellular areas (0,1); presence of brown, probably iron deposits (0,1); presence of blue, probably calcium deposits (0,1), and osseous metaplasia (0,1). FLC were defined as elongated cells, separated from vessel lumen with vesicular nucleus and one to three nucleoli. Acute inflammatory reaction was defined by the presence of neutrophilic granulocytes. Chronic inflammatory reaction was defined by the presence of round cells without taking into consideration further subclasses such as lymphocytes, plasma cells, monocytes and histiocytes. HPF = 0.26 mm^2^, MPF = 0.91 mm^2^, LPF^ = ^3.2 mm^2^. Two experienced observers (one pathologist and one nephrologist) blinded to the specimen’s diagnosis evaluated each section in two independent rounds.

The intra-observer variability of 20 histological variables was studied in a two level classification and a four level classification by two observers blinded to diagnosis. The intra-observer variability of a two level classification versus a four level classification system showed better kappa values. A four level classification was not suitable for all investigated variables because some findings were only classified as absent or present. Using a two level classification, the mean kappa value of the intra-observer variability increased form 0.55±0.12 to 0.70±0.07 (p = 0.002). The intra- and inter-observer variability is given in Table 1. For most morphological findings, the intra- and inter-observer variability was moderate (kappa value >0.4) or good (kappa values >0.6). Parameters with kappa values <0.4 were either not considered for further evaluation or a consensus evaluation was done.

**Table 1:** Intra- and inter-observer variability of 20 histological variables (two level classification).

Variable	Intra- observer variabilityMean kappa	Inter- observer variabilityMean kappa
Fibrosis	0.56	0.51
FLC	0.63	0.12
Exudation	0.72	0.75
Mesothelial denudation	0.75	0.73
Acellular areas	0.69	0.60
Cellularity	0.63	0.68
Cellularity- variability	0.54	0.29
Vessel density	0.63	0.47
Vessel density- variability	0.40	0.26
Acute inflammation	0.82	0.41
Chronic inflammation	0.67	0.57
Vasculopathy	0.48	0.23
Hemorrhage	0.79	0.47
Fibrin deposits	0.75	0.93
Calcification	1	0
Iron deposits	0.79	0.11
Ossification	No event	No event
Mesothelial hyperplasia	No event	No event
Podoplanin vascular	0.87	0.72
Podoplanin avascular	0.88	0.12

### Statistical Analysis

Two observers blinded to the diagnosis performed the semi quantitative scoring. Variables were classified as either binary (present or absent) or ordinal. The ordinal variables were discriminated as absent, low grade, moderate grade and high grade. We compared a four level classification system with a two level classification system. Each parameter was analyzed for its intra-observer and inter-observer variability. All data were processed using the software program S-Plus (version 6.1). Comparisons between different disease groups were made using analysis of variances (ANOVA) and the Fisher-test. Statistical results with a p-value p<0.05 are considered as significant, such with p<0.01 as highly significant, and such with p<0.001 as very highly significant. To predict EPS on the basis of a given set of predictor variables we used Random Forest version 4.6-2 (R Development core, version 2.11.0) [26], a classification method which is well known for its accuracy in classifying new cases [27]. Due to the phenomenon of separation, plain logistic regression can’t be applied.

## Results

Biopsies from the peritoneum of 27 patients on PD without signs of EPS were compared to 31 patients with clinical and radiological diagnostic criteria for EPS (Table 2). Clinical data were available in 29 of 31 EPS patients. All EPS patients underwent major surgery with with peritonectomy and enterolysis (PEEL) due to bowel obstruction. All patients in the EPS group had symptoms consistent with EPS. Abdominal pain or vomiting were reported by all patients in the EPS group. Additionally, a large proportion had both symptoms. Weight loss was noted in almost all patients in this group.

**Table 2:** Imaging and clinical features of EPS patients.

*Imaging features*	
Peritoneal enhancement	15/25
Peritoneal thickening	23/25
Peritoneal calcifications	4/25
Large bowel wall thickening	6/25
Small bowel wall thickening	11/25
Adhesions of bowel loops	16/25
Signs of bowel obstruction	12/25
Fluid loculation/septation	17/25
***Clinical features***	
Bowel obstruction	
Nausea and vomiting	29/29
Loss of appetite	29/29
Loss of weight	24/29
Abdominal pain	29/29
Diarrhea	11/29
*Inflammation*	
Fever	8/29
Ascites	20/29

CT scanning was performed in 19 out of 31 patients. Most common CT findings reported to support the diagnosis of EPS were peritoneal thickening (12 of 19 patients), small bowel dilatation caused by bowel obstruction was reported in 8 of 19 patients. Other findings were calcification and ascites. Up to know, 24 of 29 EPS patients were alive (mean follow up 62.1±37.0 months). The clinical data from the patient cohort is summarized in Table 3.

**Table 3:** Clinical data of study patients.

Variable	PD – no EPS	EPS
n	27	31
Age	50.6	46.4
(years;mean ±SD)	±13.6	±12.8
Female/Male**	13/14	7/24
PD-duration in	37.6	77.5
months***	±38.0	±41.2
Peritonitis**	20 in 1014 months	55 in 2170 months
	1∶50.7	1∶39.5
Transporter status**		
High/high average	4	11
Low/low average	8	3
N.D. last 6 months	15	17
Composition of PDF		
Neutral pH	5/27	9/31
Acidic pH	8/27	12/31
Both or N.D.	14/27	10/31
Icodextrin**	2/14	17/21
	13 N.D.	10 N.D.
Diabetes	8/27	7/31
Smoker***	4/24	10/19
	3 N.D.	12 N.D.
Hypertension***	19/27	26/31
Hb	11.2	10.4
(g/dl ± SD [13]–[18])	±1.7	±3.4
Leukocytes**	7.6	9.5
(G/L ± SD [4.0–11.3])	±2.0	±3.8
Phosphate	1.8	1.6
(mmol/l [0.68–1.68])	±0.6	±0.6
Calcium	2.4	2.3
(mmol/l [1.90–2.70])	±0.2	±0.4
PTH	17.9	20.5
(pmol/l [1.1–7.3])	±17.1	±22.5
Urea-N**	58.2	39.6
(mg/dl [10]–[25])	±31.5	±18.2
Creatinine	8.1	7.3
(mg/dl [0.5–1.4])	±3.2	±2.4

PD, peritoneal dialysis; EPS, encapsulating peritoneal sclerosis; PDF, peritoneal dialysis fluid; Hb, haemoglobin; N.D., not determined; PTH, parathyroid hormone,

*** p<0.001.

** p<0.05.

* p<0.1.

Compared to the PD-group, there were significant more male than female in the EPS-group. Time on PD was longer and peritonitis rate was higher in EPS patients. As expected, there was a significant difference regarding transporter status and use of icodextrin between the two groups. None of the PD-patients without EPS had ultrafiltration failure, whereas 8 out of 31 patients in the EPS group developed ultrafiltration failure. Smokers and patients suffering from hypertension were more common in EPS. Leucocytes were higher and urea-N was lower compared to patients in the PD-group.

**Figure 1:**
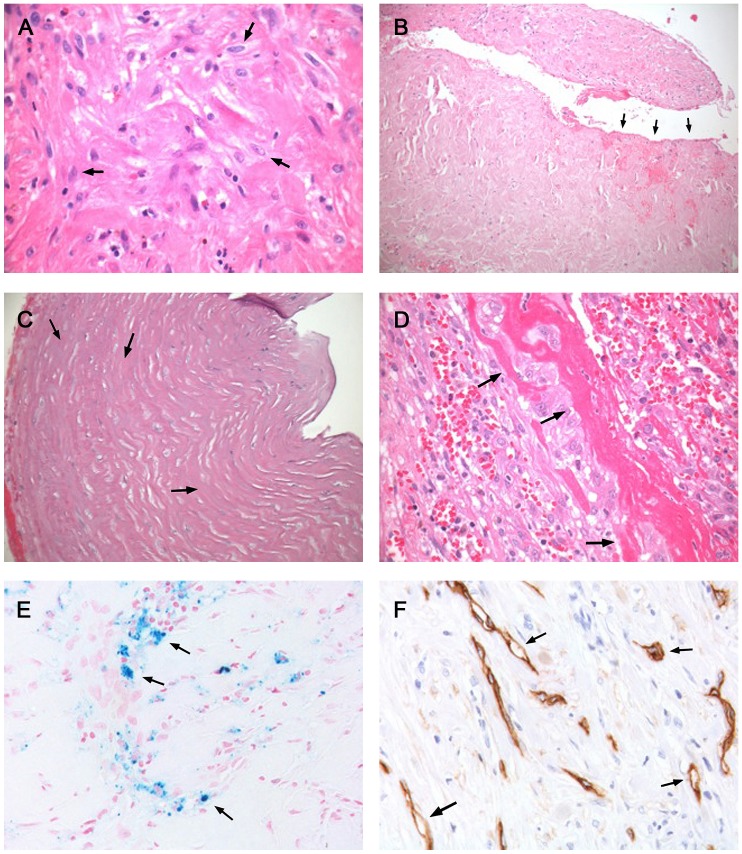
Histopathological findings in EPS compared to simple sclerosis. **A** HE staining showing an increased cellularity, round cells and fibroblast like cells (arrows). EPS, original magnification×400; **B** HE staining showing a decreased cellularity, fibrin deposits and a complete denudation of the mesothelial cell layer with fibrin exudations (arrows). EPS, original magnification×100; **C** HE staining showing a decreased cellularity with intracellular matrix (arrows), complete mesothelial denudation with fibrin exudations. EPS, original magnification×200; **D** HE staining showing an increased cellularity, hemorrhage, round cells, fibroblast like cells and fibrin deposits (arrows). EPS, original magnification×400; **E** Fe staining showing vessels, intraluminal erythrocytes and Fe deposits (arrows). EPS, original magnification×400; **F** D2–40 stained section showing podoplanin positive cells associated to vessels (arrows). EPS, original magnification×400.

**Figure 2:**
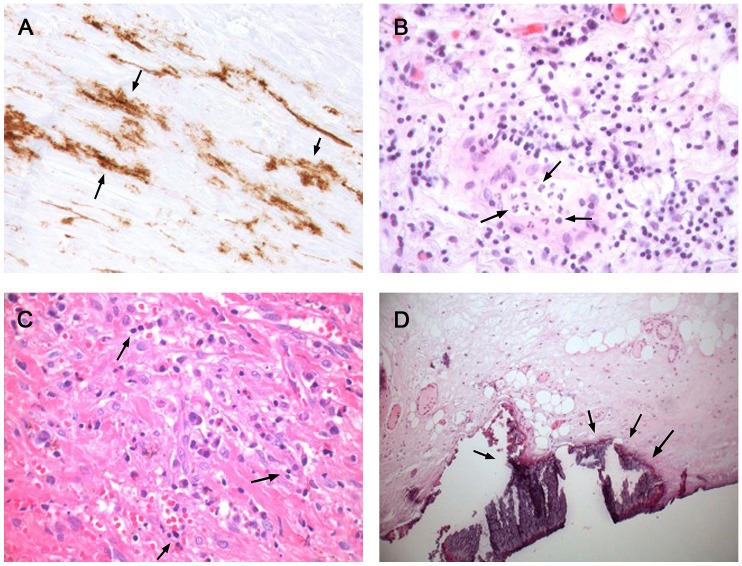
Histopathological findings in EPS compared to simple sclerosis. **A** D2–40 stained section showing podoplanin positive cells not associated to vessels (arrows). EPS, original magnification×400; **B** HE staining showing acute and chronic inflammation with round cells and neutrophils (arrows). EPS, original magnification×400; **C** HE staining showing fibroblast like cells, eosinophils, plasma cells and round cells (arrows). EPS, original magnification×400; **D** HE staining showing vasculitis, round cells and calcium deposits (arrows). EPS, original magnification×100.

**Figure 3:**
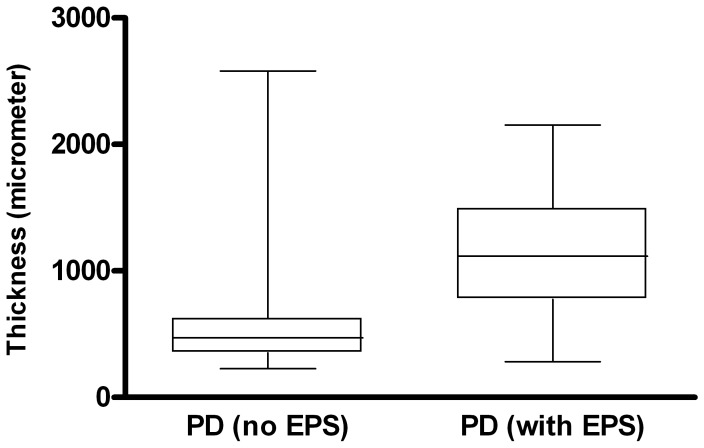
Thickness of the fibrosis zone in the submesothelial cell layer in PD patients and patients on PD with EPS. p = 0.031; range PD group 227–2581 µm; range EPS group 281–2150 µm.

The following findings were significantly more common in EPS than in PD-patients without EPS (Table 4): fibroblast-like cells (FLC) (p<0.0001), mesothelial denudation (p<0.0001), decreased cellularity (p = 0.008), fibrin deposits (p<0.03), positive iron staining (p = 0.05) and immunohistochemistry for podoplanin vascular (p<0.0001) and podoplanin avascular (p<0.0001) (Figure 1 and 2). A first semi quantitative approach, regarding the degree of fibrosis showed no significant difference. A second quantitative consensus analysis with optimized tissue engineering showed a significant difference between EPS and PD: 602,9 µm vs. 1132,5 µm; p = 0.0031 (Figure 3). Due to the methodological effort and the need for consensus analysis to increase the reproducibility, we excluded fibrosis from further analysis.

**Table 4:** Frequency of histologic findings in EPS compared to simple sclerosis.

Variable	PD - no EPS	EPS	p
*Fibrosis*	20/27	26/31	0.32
*FLC (consensus)*	5/27	18/31	<0.0001
*Exudation*	2/27	8/31	0.089
*Mesothelial denudation*	17/27	31/31	<0.0001
*Acellular areas*	11/27	23/31	0.008
*Cellularity*	7/27	14/31	0.17
*Cellularity- variability*	5/27	7/31	0.76
*Vessel density*	11/27	16/31	0.61
*Vessel density- variability*	8/27	7/31	0.54
*Acute inflammation*	2/27	10/31	0.23
*Chronic inflammation*	8/27	16/31	0.11
*Vasculopathy (consensus)*	7/27	11/31	0.40
*Hemorrhage*	3/27	4/31	1.00
*Fibrin deposits*	0/27	6/31	0.03
*Calcification*	0/27	3/31	-
*Iron deposits*	1/27	7/31	0.05
*Ossification*	No event	No event	-
*Mesothelial hyperplasia*	No event	No event	-
*Podoplanin vascular*	4/27	24/31	<0.0001
*Podoplanin avascular*	7/27	23/31	<0.0001

Using all predictor variables as given in Table 4 we trained the classification method Random Forest to categorize future cases (Table 5). Podoplanin vascular and avascular were taken together (p<0.0001), FLC (p<0.0001), mesothelial denudation (p = 0.0005), calcification (p = 0.0026), acellular areas (p = 0.0094), fibrin deposits (p = 0.0336) showed up as significantly important predictor variables. Estimated misclassification error rate when classifying new cases turned out to be 14%.

**Table 5:** Classification of EPS and non-EPS cases by using random forests.

Predictor	Importance	p
Podoplanin	4.6161	<0.0001
FLC	3.8859	<0.0001
Denudation	3.3190	0.0005
Calcification	2.7935	0.0026
Acellular areas	2.3493	0.0094
Fibrin deposits	1.8297	0.0336
Vasculopathy	1.5955	0.0553
Acute inflammation	1.3537	0.0879
Iron deposits	1.2978	0.0972
Cellularity	0.1393	0.4446
Vessel density	0.0240	0.4904
Exudation	−0.1022	0.5407
Fibrosis	−0.2180	0.5863
Chronic inflammation	−0.3962	0.6540
Hemorrhage	−0.7729	0.7802

The first column shows a scaled measure of the relative importance of each predictor variable. The second column lists the p-values related to one-sided z-tests. The appearance of the predictor variables was ordered according to decreasing importance. The estimated misclassification error rate for new cases is 14%.

## Discussion

There are significant morphological differences between peritoneal biopsies of PD-patients compared to PD-patients with EPS. Our study is the first standardized approach to define histological criteria in the diagnosis of EPS.

We investigated histological parameters introduced in previous studies and combined them with further promising parameters [20]–[22]. To keep these results reproducible in daily practice, we calculated the intra- and inter-observer variability (two- and four-level) and excluded kappa values below 0.4. To keep histological work-up simple, we used HE-staining and one single immunohistochemical staining (podoplanin). Podoplanin was found to be a good marker for lymphatic endothelial cells, but it is also expressed by peritoneal mesothelial cells [28]. As podoplanin can bind chemokines, it may modulate the inflammatory milieu, and therefore might be involved in the injury process of both simple sclerosis and EPS [29]. A previous study of our research group demonstrated that podoplanin might be a suitable marker to discriminate between simple sclerosis and EPS [16].

The chosen statistical method in this study is Random Forest. Due to empty cells in cross tabulation and co-linearity of variables plain multiple logistic regression can’t be applied. Random Forest is a classification system, which consists of many different decision trees. Each tree gives a classification and votes for a class. The forest chooses the classification having the most votes. Using Random Forest it is possible to calculate the importance of each histological parameter. Therefore, it is the optimal statistical method for a web-based database. Even small centers with only few EPS cases will have the opportunity to benchmark their histological findings and to calculate the misclassification error. The method allows to calculate the probability of a new case to be either EPS or simple sclerosis. For example a case with fibrosis (1), FLC (1), exudation (0), increased vessel density (0), increased cellularity (0), acute inflammation (1), chronic inflammation (0), heamorrhage (0), fibrin deposits (0), Fe deposits (0), calcification (1), mesothelial denudation (1), acellular areas (1), vasculopathy (0) and podoplanin staining (1) has a EPS probability of 0.96. We expect that the misclassification error of 14% in our study will improve with every new case added to the database.

Mesothelial denudation, calcifications and acute and chronic inflammation were previously described [18], [20], [22]. Fibrin deposits, previously reported as a landmark of EPS, were found in 6 out of 31 EPS cases but in none of the PD-group. The fibrin deposition could be linked to decreased fibrin clearance by peritoneal mast cells [30].

Decreased cellularity, with an increased amount of intracellular matrix, is a new finding. An explanation for this finding in our cohort could be the late stage of the disease [30].

FLCs are indicative for EPS. Previous studies identified this cell type as fibroblasts, activated fibroblasts or myofibroblasts [16], [31]. Without using immunohistochemistry, further differentiation of this cell type is not possible. To keep analysis simple, all cells with this fibroblast-appearance were characterized as fibroblast-like cells (FLC).

Angiogenesis, often described as a typical finding in EPS, is difficult to reproduce, because the variability of the number of vessels in the same slide is high and most of the detected vessels are probably lymphatic vessels [15], [16], [20].

In our study we performed a semi quantitative analysis of the extend of fibrosis and found no significant difference between EPS and PD. A second, quantitative consensus approach followed by optimized tissue engineering showed a significant result. Remarkably, the range of fibrosis was very much the same in the two groups and underlines the problem of a reliable analysis. Due to the complex methodology and the low reproducibility, fibrosis was excluded from further analysis. Previous studies regarding histological parameters in EPS and PD measured the submesothelial cell layer, the compact zone, the degenerated layer thickness and the thickness of sclerosis. Details about tissue engineering were not given and definitions and details regarding the measured variables are unclear [21], [22]. Further multicenter approaches with a consensus regarding these variables are needed.

In our cohort, calcifications were rare and ossifications were absent. If calcification was present, it was highly indicative for EPS.

Several of the analyzed parameters occur primarily in EPS and are uncommon in PD-patients. Few EPS patients exhibit these morphological changes, which lead us to conclude that a combination of histological criteria is necessary to increase specificity and sensitivity.

The combination of clinical criteria [7], [32], [33], radiological scores [6], [11], [13] and standardized histological criteria, will enable nephrologists and pathologists to further increase sensitivity and specificity in the diagnosis of EPS. Recently implemented minimally invasive procedures are setting the stage to facilitate tissue retrieval with minimal damage to the peritoneal membrane [34], [35].

Our study is a first standardized approach towards histological criteria for EPS. Several limitations of the study have to be addressed. Our institution is a referral center for PD patients. Therefore, in patients referred from other centers, measurement of membrane function is not standardized. Recent membrane function tests are sometimes not available in all patients at the time of diagnosis. Additionally, due to the difference in time on PD between the two groups it is possible that some of the histological features in the EPS group could reflect non-specific membrane damage from PD rather than EPS itself.

In conclusion, we introduced standardized histological criteria for EPS and a statistical method, which facilitates to discriminate between simple sclerosis and EPS. To confirm these findings, further studies in unrelated cohorts will examine histological specimens of additional EPS patients.
